# An explainable machine learning model to solid adnexal masses diagnosis based on clinical data and qualitative ultrasound indicators

**DOI:** 10.1002/cam4.7425

**Published:** 2024-06-24

**Authors:** Annarita Fanizzi, Francesca Arezzo, Gennaro Cormio, Maria Colomba Comes, Gerardo Cazzato, Luca Boldrini, Samantha Bove, Michele Bollino, Anila Kardhashi, Erica Silvestris, Pietro Quarto, Michele Mongelli, Emanuele Naglieri, Rahel Signorile, Vera Loizzi, Raffaella Massafra

**Affiliations:** ^1^ Laboratorio Biostatistica e Bioinformatica I.R.C.C.S. Istituto Tumori ‘Giovanni Paolo II’ Bari Italy; ^2^ Gynecologic Oncology Unit IRCCS Istituto Tumori “Giovanni Paolo II” Bari Italy; ^3^ Department of Precision and Regenerative Medicine – Ionian Area University of Bari “Aldo Moro” Bari Italy; ^4^ Interdisciplinar Department of Medicine University of Bari “Aldo Moro” Bari Italy; ^5^ Section of Molecular Pathology, Department of Emergency and Organ Transplantation University of Bari “Aldo Moro” Bari Italy; ^6^ Fondazione Policlinico Universitario “A. Gemelli” IRCCS Italy; ^7^ Department of Obstetrics and Gynecology, Division of Gynecologic oncology, Skåne University Hospital and Lund University Faculty of Medicine, Clinical Sciences Lund Sweden; ^8^ Medical Oncology Unit, IRCCS Istituto Tumori Giovanni Paolo II Bari Italy

**Keywords:** gynecological ultrasound, machine learning, ovarian cancer, precision medicine, solid adnexal masses

## Abstract

**Background:**

Accurate characterization of newly diagnosed a solid adnexal lesion is a key step in defining the most appropriate therapeutic approach. Despite guidance from the International Ovarian Tumor Analyzes Panel, the evaluation of these lesions can be challenging. Recent studies have demonstrated how machine learning techniques can be applied to clinical data to solve this diagnostic problem. However, ML models can often consider as black‐boxes due to the difficulty of understanding the decision‐making process used by the algorithm to obtain a specific result.

**Aims:**

For this purpose, we propose an Explainable Artificial Intelligence model trained on clinical characteristics and qualitative ultrasound indicators to predict solid adnexal masses diagnosis.

**Materials & Methods:**

Since the diagnostic task was a three‐class problem (benign tumor, invasive cancer, or ovarian metastasis), we proposed a waterfall classification model: a first model was trained and validated to discriminate benign versus malignant, a second model was trained to distinguish nonmetastatic versus metastatic malignant lesion which occurs when a patient is predicted to be malignant by the first model. Firstly, a stepwise feature selection procedure was implemented. The classification performances were validated on Leave One Out scheme.

**Results:**

The accuracy of the three‐class model reaches an overall accuracy of 86.36%, and the precision per‐class of the benign, nonmetastatic malignant, and metastatic malignant classes were 86.96%, 87.27%, and 77.78%, respectively. Discussion: SHapley Additive exPlanations were performed to visually show how the machine learning model made a specific decision. For each patient, the SHAP values expressed how each characteristic contributed to the classification result. Such information represents an added value for the clinical usability of a diagnostic system.

**Conclusions:**

This is the first work that attempts to design an explainable machine‐learning tool for the histological diagnosis of solid masses of the ovary.

## 1 INTRODUCTION

Accurately characterizing newly diagnosed adnexal lesions is crucial for determining the appropriate treatment approach. In clinical practice, a gynecological ultrasound examination is commonly considered the standard initial imaging investigation for evaluating adnexal tumors.^1^ To ensure consistency and uniformity in the quality, description and evaluation of ultrasonography performed at different centers, and to enhance diagnostic accuracy, the International Ovarian Tumor Analysis (IOTA) group published a consensus paper in 2000. Such a paper aimed to establish standardized terms and definitions for describing adnexal lesions.^2^ Based on the analysis of morphological features, the IOTA group introduced a qualitative classification system consisting of six categories. Among these, a solid tumor was defined as a lesion in which solid components make up 80% or more of the tumor.^2^

A solid adnexal tumor detected through ultrasound can be either a benign tumor, an invasive carcinoma or an ovarian metastasis. Evaluating these lesions can be challenging, but it is a crucial step in ensuring the patient receives appropriate management for their condition. Patients with masses that raise suspicion for primary ovarian malignancy should be referred to a gynecological oncology center to receive specialized care.^3^ Patients with lesions that are likely benign can be followed conservatively.^4^ On the other hand, in patients with suspected adnexal metastasis, second‐level instrumental evaluations should be requested to identify the neoplasm's origin and refer the patient to the appropriate specialist.^5^

Recent scientific studies demonstrated how machine learning techniques applied to clinical data provide added value in exploiting them to fulfill predictive tasks,^6^ thus contributing to the definition of support systems for clinical and therapeutic decisions that can help clinicians answer crucial unmet clinical needs. However, machine learning techniques can often be considered as black‐boxes due to the difficulty of understanding the decision‐making process used by the algorithm to obtain a specific result. For this purpose, Explainable Artificial Intelligence (XAI) has been introduced, whose main aim is to overcome the black‐box concept and define intelligible tools that can be used in clinical practice in a more informed manner.^7^, ^8^, ^9^, ^10^, ^11^, ^12^ Furthermore, Explainable techniques, such as SHapley Additive exPlanations (SHAP), based on the calculation of Shapley values,^13^ enable to visually show how the machine learning model made a specific decision. In this vein, in this work, we propose an explainable machine learning model trained on clinical characteristics and qualitative ultrasound indicators to predict solid adnexal masses diagnosis, that is, whether it is a benign, nonmetastatic malignant or metastatic malignant tumor. The classification performance of the proposed machine learning model was compared with ADNEX and Simple Rules tools and with our expert radiologist's subjective assessment.

## 2 MATERIALS AND METHODS

### 2.1 Materials

The study was conducted according to the guidelines of the Declaration of Helsinki and approved by the Ethics Committee of the Azienda Ospedaliera Policlinico Consorziale‐University of Bari, Italy (protocol code n. 6398/2020).

In this retrospective observational study, we analyzed consecutive patients with solid adnexal masses who were followed in a tertiary center from May 2020 to December 2022.

All patients received a preoperative transvaginal or transrectal ultrasound examination and additional transabdominal ultrasound when necessary, according to IOTA classification. Ultrasound examinations were performed by a specialized ultrasound examiner with a 5.0–9.0 MHz vaginal probe or 3.5–5.0 MHz abdominal probe. All ultrasound reports and images were available for analysis.

We collected clinical data and qualitative ultrasound indicators of 110 consecutive patients affected by solid ovarian tumors.

### 2.2 IOTA Models—ADNEX and simple rules

To create a tool to aid clinicians in the ultrasound assessment of adnexal masses, the IOTA group devised “simple rules” that can be used to evaluate a mass based on the identification of five benign and five malignant ultrasound features.^14^ These rules can be applied to approximately 80% of adnexal masses, while the remaining cases are categorized as inconclusive. Regarding solid tumors, according to this tool, purely solid masses with irregular borders were found to be almost always malignant, regardless of their size or level of vascularity.^14^

Simple Rules are indeed a useful tool for distinguishing between benign and malignant adnexal masses. However, they do not provide information on the likelihood of a metastatic lesion. Additionally, Simple Rules do not allow clinicians for the estimation of a malignancy risk percentage.^15^

Afterward, the IOTA group developed the Assessment of different NEoplasias in the adneXa (ADNEX) model. This model is the first risk assessment tool that distinguishes between benign and four subtypes of malignant ovarian tumors: borderline tumors, stage I cancer, stage II–IV cancer, and secondary metastatic cancer.^16^

In this study, the diagnostic accuracy of these two IOTA models commonly used in clinical practice, ADNEX and Simple Rules, was evaluated on our real‐life sample of patients with ultrasound findings of solid adnexal lesions.

### 2.3 Machine learning classification model

**FIGURE 1:**
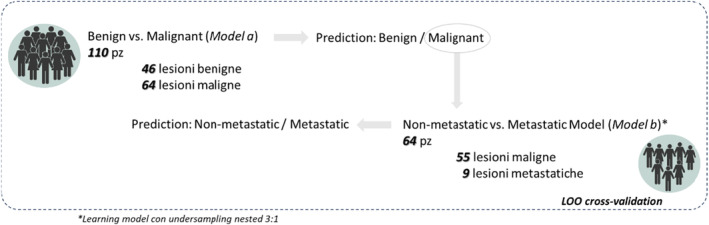
Graphical representation of waterfall model for solving a three‐class classification task. A first model was trained and validated to solve the benign versus malignant (*model a*), and a second model was trained to solve the metastatic versus nonmetastatic (*model b*). For the *model b*, the training set within the different leave one out (LOO) cross‐validation fold was undersized with a 3:1 ratio in the training phase of the classifi‐cation model.

The diagnostic task to be solved is a three‐class problem, such as solid tumors have been distinguished in benign, malignant, and metastatic tumors. For this reason, the classification model proposed in this work is a *waterfall* model: a first model was trained and validated to discriminate benign versus malignant (*model a*), a second model was trained to distinguish nonmetastatic versus metastatic (*model b*) which occurs when a patient is predicted to be malignant lesion by the *model a* (Figure 1). Specifically, *model a* was trained retrospectively on the entire sample, thus including, in addition to the cases of benign lesions as the first class, and both the cases of metastatic malignant and nonmetastatic malignant lesions as the second class. Therefore, *Model b*, was trained on the subsample of patients with malignant lesion thus including cases of metastatic and nonmetastatic lesions as two different classes.

**FIGURE 2:**
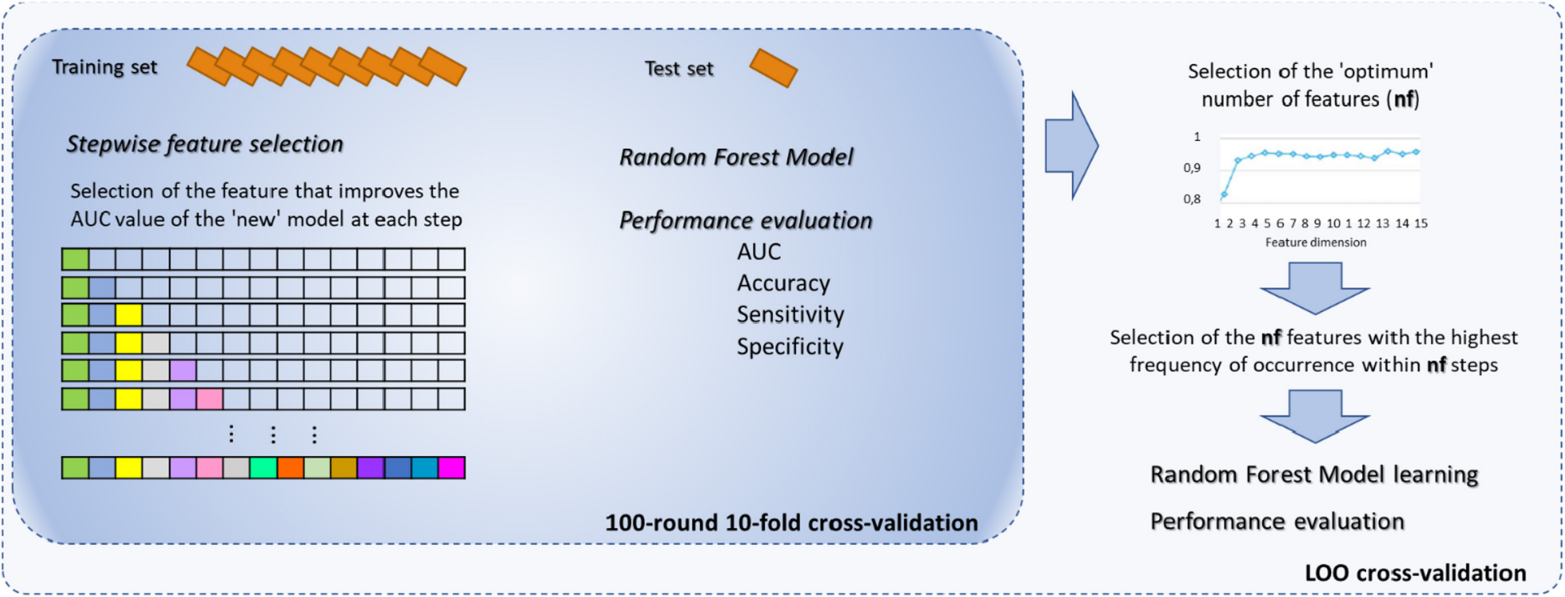
General workflow of the learning binary classification models. Both *model a* and *model b* consisted of a first phase of feature selection using a stepwise forward selection algorithm performed on 100 10‐fold cross‐validation rounds. Subsequently, the optimal number of features at which the highest median AUC was observed was identified. Then, the subset of features is those features that showed the highest frequency of occurrence in the 100 10‐fold cross‐validation rounds. Finally, the performances in LOO cross‐validation were evaluated using the subset of features thus selected for each specific model.

The classification model framework for both classification problems was analogous and represented in Figure 2. Firstly, a stepwise feature selection procedure was implemented. This is a wrapped method which follows a search approach of optimal features by evaluating different possible combinations of features according to a specific assessment criterion. Specifically, we performed a stepwise forward feature selection based on Random Forest (RF) classifier, an ensemble machine learning classifier that generally performs well, overcoming the over‐fitting issue.^17^ Furthermore, RF provides an embedded method for feature selection: it takes advantage of its feature selection process and performs classification simultaneously. In our work, we used the decrease in Gini impurity when a feature is chosen to split a tree node and a standard configuration of RF with 100 trees and 20 features (as described in^18^) randomly selected at each split.

Moreover, to avoid over‐fitting, we have fixed a small number of observations per tree leaf (three). Other state‐of‐the‐art machine learning classifiers were evaluated but did not lead to a significant improvement in performance. To avoid burdening the discussion of the work, we neglected their performance.

The forward sequential selection algorithm identifies a subset of the features that best predict the desired result by sequentially adding at each step the feature that increases the performance of the RF classifier in terms of Area Under the Curve value on the training set of cross‐validation. Moreover, even to reduce the overfitting of the model, this procedure was implemented on 100 ten‐fold cross‐validation rounds. Then, we evaluated the ‘optimal’ number of feature sets (*nf*) at which the highest median AUC value of the model was observed over the 100 10‐fold cross‐validation rounds. Therefore, we selected the feature set most frequently selected within the optimal number of features thus defined in the different evaluation rounds.

As anticipated, the above analysis workflow is similar for *models a a*nd *b*. Still, it should be emphasized that due to the strong imbalance of the metastatic versus nonmetastatic classes, to train model b the training set within the different LOO cross‐validation fold was undersized with a 3:1 ratio in the training phase of the classification model.^19^ Finally, it is emphasized that missing clinical attribute values were treated according to the predictive value imputation method by replacing missing values with the average of the attribute observed in the training set.^20^

The classification performances of the two binary models were validated on LOO scheme and evaluated in terms of AUC of the receiver operating characteristic (ROC) curve, accuracy, sensitivity, and specificity calculated by identifying the optimal threshold by Youden's index on ROC curves.^21^

The classification performances of multiclass model were evaluated in terms of overall accuracy and per‐class sensitivity. Per‐class sensitivity was calculated for a specific one class versus all remaining classes.

All the analyses were performed using MATLAB R2022a (Mathworks, Inc., Natick, MA, USA) software.

### 2.4 The explainable algorithm

At the end of the performance evaluation of each of the two trained models, that is, *model a* and *model b*, we implemented a well‐known Explicable Artificial Intelligence (XAI) technique to clarify how the classifier returned a decision for each given patient. Specifically, we adopted a cutting‐edge local explanation algorithm, SHAP,^22^ that is a local model‐agnostic approach using only a classifier's input and output. Specifically, regardless of the rules that the classifier model has learned about the data, the SHAP algorithm estimates Shapley values, that are the contribution of each feature value on predictions referred to each individual test sample by evaluating each marginal contribution with respect to all the features considered together.^22^, ^23^ The absolute SHAP value of a feature is greater as greater its weight in defining the classification score.

In our case of study, a positive Shapley value referred to a specific feature indicates that this feature contributed to increasing the probability that the solid lesions of the ovary was either malignant in *model a* or metastatic in *model b*. In contrast, a negative Shapley value indicates that this feature reduced the same probability.

A graphical representation of these contributions referring to the individual patient could help clinicians to evaluate the suggestion proposed by the classification model.

## 3 RESULTS

### 3.1 Characteristics of the collected sample

Table 1 summarizes the characteristics of the analyzed samples. For each patient, clinical characteristics (age at diagnosis, personal history of breast cancer, parity, menopausal status, family history of cancer, CA125) were collected as well as data about US examinations according to IOTA classification (bilaterality, side, origin of the lesion, largest diameter of lesion, shadows, ovarian crescent sign, color score, ascites, free fluid).

**TABLE 1:** Characteristics of the 110 patients analyzed in the study.

Characteristics	Distribution
Diagnosis	
Benign (Abs; %)	46; 41.81%
Nonmetastatic malignant (Abs; %)	55; 50.00%
Metastatic malignant (Abs; %)	9; 8.18%
Age at diagnosis	
Median (1th quartile; 3th quartile)	54.50 (47.00; 65.75)
Nan (Abs; %)	
Personal history of breast cancer	
Yes (Abs; %)	31; 28.18%
No (Abs; %)	79; 71.82%
Nan (Abs; %)	–
Parity	
0 (Abs; %)	34; 30.91%
1 (Abs; %)	27; 24.55%
Multiparous Nan	48; 43.63% 1; 0,91%
Menopausal status	
Yes (Abs; %)	72; 65.45%
No (Abs; %)	38; 35.55%
Nan (Abs; %)	–
Family history of cancer	
No (Abs; %)	69; 62.73%
Breast cancer (Abs; %)	13; 11.82%
Ovarian cancer (Abs; %)	21; 19.09%
Breast and ovarian cancer (Abs; %)	7; 6.36%
Nan	–
CA125 (U/mL)	
Median (1th quartile; 3th quartile)	60.80 (75.50; 1135.25)
Nan (Abs; %)	
Bilaterality	
Yes (Abs; %)	31; 28.18%
No (Abs; %)	79; 71.82%
Nan (Abs; %)	–
Side	
Right (Abs; %)	48; 43.64%
Left (Abs; %)	48; 43.46%
Central (Abs; %)	14; 12.73%
Nan (Abs; %)	–
Origin of the lesion	
Ovarian (Abs; %)	98; 89.09%
Other (salpinx, paraovarian, paratubal) Uncertain (Abs; %)	6; 5.45% 6; 5.45%
Largest diameter of lesion (mm)	
Median (1th quartile; 3th quartile)	59.50 (46.50; 121.25)
Nan (Abs; %)	–
Shadows	
Yes (Abs; %)	45; 40.91%
No (Abs; %)	65; 59.09%
Nan (Abs; %)	–
Ovarian crescent sign	
Yes (Abs; %)	33; 30.00%
No (Abs; %)	72; 65.45%
Normal ovary (Abs; %)	3; 2.73%
Uncertain (Abs; %)	2; 1.81%
Nan (Abs; %)	–
Color Score	
No flow (Abs; %)	24; 21.82%
Minimal flow (Abs; %)	18; 16.36%
Moderate flow (Abs; %)	17; 15.45%
Very strong flow (Abs; %)	51; 46.36%
Nan (Abs; %)	–
Ascites	
Yes (Abs; %)	30; 27.27%
No (Abs; %)	80; 73.73%
Nan (Abs; %)	–
Free fluid	
Yes (Abs; %)	54; 49.09%
No (Abs; %)	56; 50.09%
Nan (Abs; %)	–

Forty‐six (41.82%) lesions were benign, whereas 64 (58.18%) were malignant, of which 9 (14.06%) were metastatic.

### 3.2 Accuracy ADNEX, simple rules on a real‐life sample and subjective assessment

On the collected sample, the real‐life performance of two well‐known diagnostic tools, such as ADNEX and Simple Rules, were evaluated for the histological outcome of the surgical sampling. In addition, the classification performance of the operator (hereinafter called subjective assessment) was also evaluated.

Concerning the binary problem (benign vs. malignant), the best performances in terms of overall Accuracy are achieved by the Simple Rules, followed by Subjective assessment, which shows an overall accuracy of 93.63% (Table 2a). However, it should be emphasized that the Simple Rules do not provide an answer for 12 cases (10.91%) of the sample considered. Specifically, they are eight benign, three nonmetastatic malignant, and one metastatic malignant tumors, respectively. Thus, the accuracy of the ADNEX tool reached a value of 80.00% with a specificity of 54.34%.

**TABLE 2:** Classification performance of ADNEX, simple rules, and subjective assessment. The indicated values are reported in percentage terms.

(a) Benign versus malignant classification task			
	Accuracy	Sensitivity	Specificity
ADNEX	80.00	98.44	54.34
Simple rules^a^	94.90	98.33	89.13
Subjective assessment	93.63	96.88	89.47

(b) Benign versus nonmetastatic malignant versus metastatic malignant classification task				
	Accuracy	Acc. benign	Acc. nonmetastatic	Acc. metastatic
ADNEX	62.73	54.35	67.27	77.78
Subjective assessment	86.36	89.13	90.90	44.44

^a^ Percentages calculated net of the 12 cases (10.91%) of the sample considered for which the instrument did not express a diagnosis, therefore deemed unclassifiable.

Regarding the three‐class problem (benign vs. nonmetastatic malignant vs. metastatic malignant), the operator's performance (subjective assessment) achieved a value of 86.36%, while those of ADNEX was 62.73%. Specifically, the ADNEX tool shows an accuracy of the metastatic class of 44.44% (Table 2b).

### 3.3 Classification performances

The waterfall model developed to solve the three‐class diagnostic problem of solid adnexal masses provides the training of two binary models independently, which are concatenated in the prediction phase. Nonetheless, the performances of the single models evaluated in a LOO validation scheme are reported below, followed by the model's performances on the three‐class problem.

The analysis workflow included a feature selection phase through a stepwise selection procedure inserted into 100 10‐fold cross‐validation rounds, following which the optimal number of features was selected, which maximized the average value of the AUC. Therefore, the features most frequently selected by stepwise procedure within the identified optimal number were used to train the final models validated in the LOO scheme. Finally, it should be underlined that in phase the cutoff identified b’ Youden's index to binarize the classification output of model a and *model b* was respectively 0.48 and 0.15, that is, that a patient with a classification score of *model a* higher at 0.48 was classified as malignant, and a patient with model classification score b (when applied) was classified as malignant if his classification score was greater than 0.15. The particularly low cutoff of *model b* reflects the imbalance of the two on which the model was trained.

**FIGURE 3:**
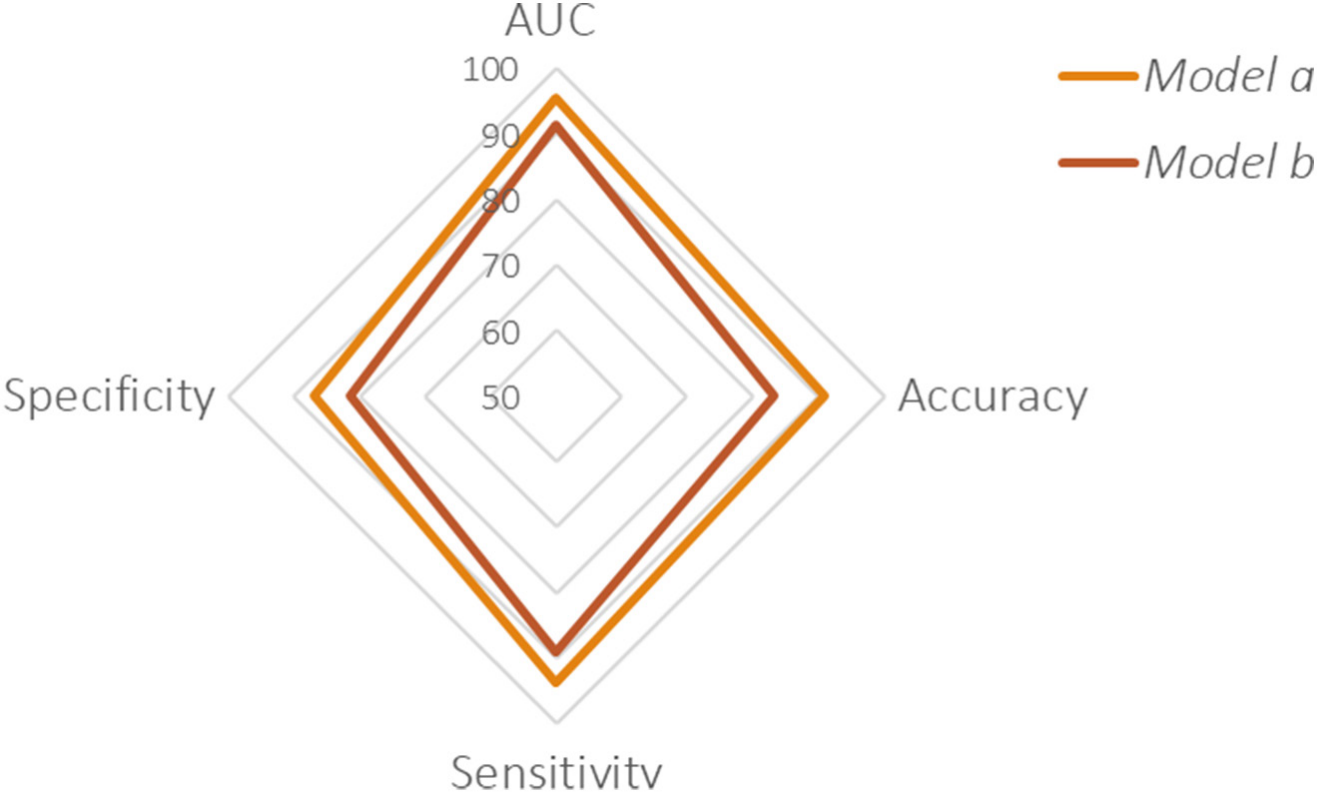
Performances classification related to binary *model a* (benign vs. malignant) and *b* (nonmetastatic malignant vs. metastatic malignant). Both models achieve highly performing performances.

Concerning the classification *model a* (benign vs. malignant), 13 characteristics were selected, that is, Color Score, CA125, Shadows, Ascites, largest diameter of lesion, personal history breast cancer, age at diagnosis, parity, ovarian crescent sign, menopausal status, tumor side, family history of cancer, and origin of the lesion. The classification performances evaluated in terms of AUC, accuracy, sensitivity, and specificity settled at 95.52%, 90.91%, 93.75%, and 86.96%, respectively (Figure 3). *Model a* outperformed the accuracy of the ADNEX tool and it is comparable to the subjective assessment, but not that of the Simple Rule tool. However, it should be underlined the simple rules did not express an evaluation in approximately 10% of cases.

Regarding classification *model b* (nonmetastatic malignant vs. metastatic malignant), 8 features were selected, that is, CA125, age at diagnosis, largest diameter of lesion, free fluid, personal history of breast cancer, family cancer history, parity of cancer and menopausal status The model's performances evaluated in LOO scheme were still highly performing (91.36, 83.33, 88.89, 81.48, respectively). Such performances have been calculated on the sub‐sample patients with malignant tumor consisting of metastatic and nonmetastatic cases (Figure 3).

**FIGURE 4:**
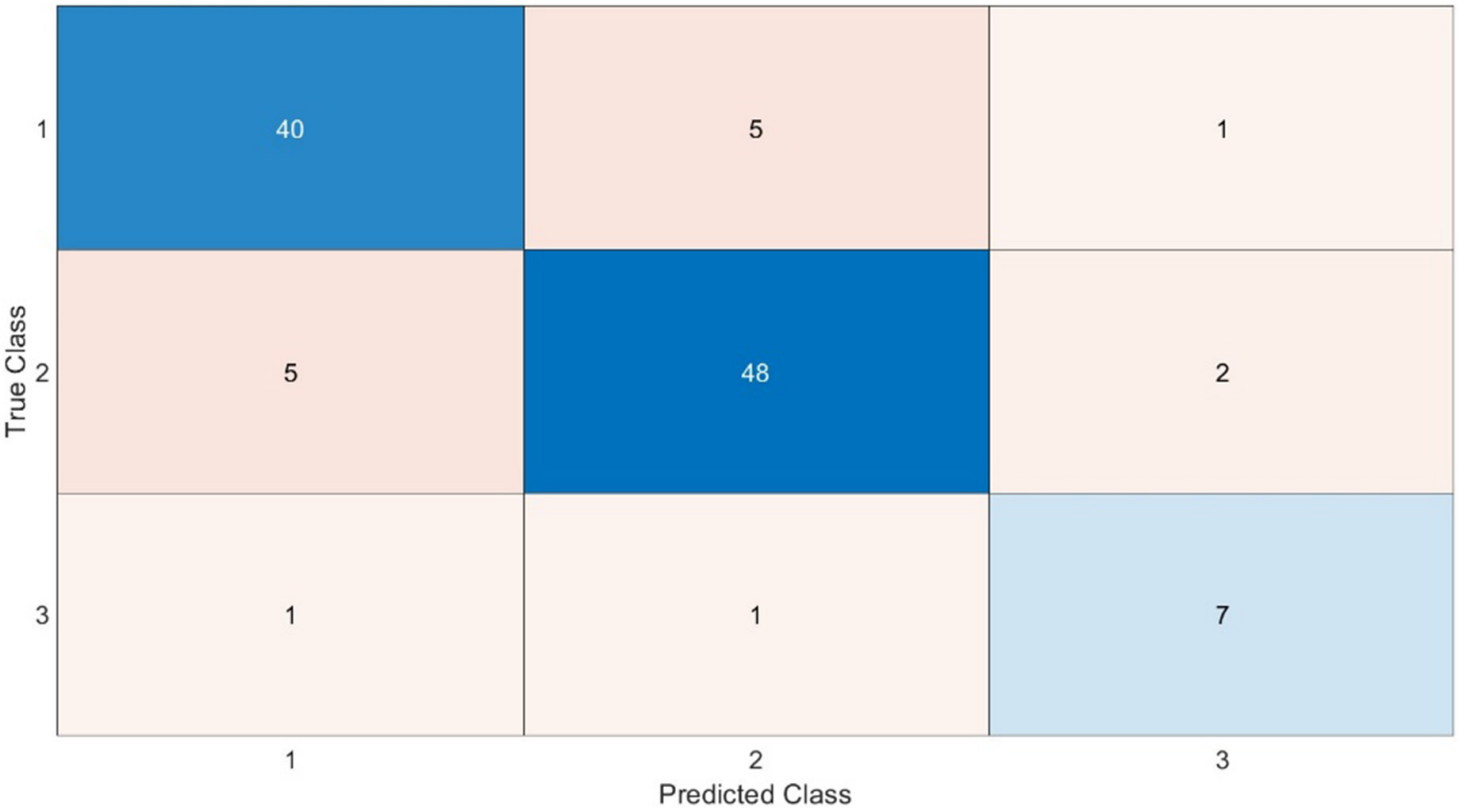
Confusion matrix of the multiclass model.

Figure 4 shown the confusion matrix of the multiclass model. By concatenating the predictions in LOO of the binary models, the accuracy of the three‐class model reaches an overall accuracy of 86.36%. Specifically, the precision per‐class of the benign, nonmetastatic malignant, and metastatic malignant classes were 86.96%, 87.27%, and 77.78%, respectively. The confusion matrix shows that the major uncertainties of the proposed multiclass model concern discrimination between benign and nonmetastatic malignant lesion. Our three‐class model outperformed the ADNEX accuracy evaluated on a real‐life studied sample (62.73%) and is still comparable with that of subjective assessment (86.36%). However, it should be emphasized that the proposed model recognizes the metastatic class more accurately than the operator's judgment, albeit losing about 3 percentage points in the other two classes.

### 3.4 Explainable machine learning model

In order to provide clinicians with a diagnostic white‐tool, we have implemented the pioneering approach of XAI. We computed Shapley's values at the local patient level for both *models a* and *b*.

**FIGURE 5:**
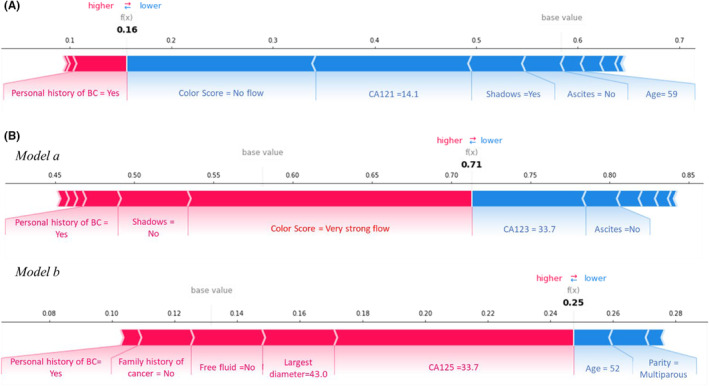
Examples of explicable outcomes of correctly classified cases. Examples of correct classification of patients with benign lesions (A) and malignant metastatic lesions (B).

**FIGURE 6:**
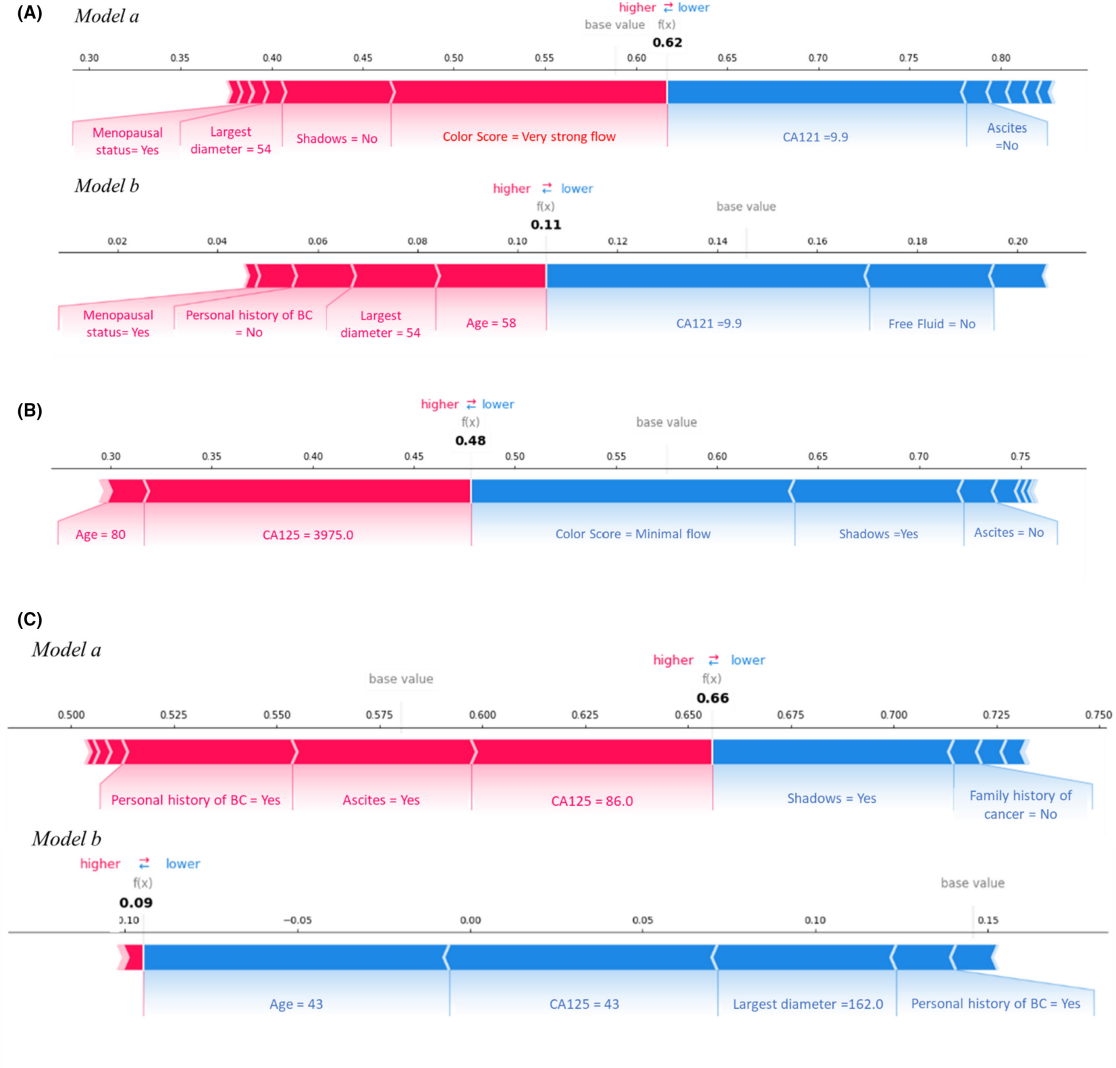
Examples of explicable outcomes of misclassified cases. Examples of misclassifications of a patient with a benign lesion (A), a malignant lesion (B), and (C) a metastatic lesion.

Figures 5 and 6 show the graphical representation of how the features used to train the two models contributed to achieving the classification score for a specific patient. In these representations, only the characteristics with the highest values in absolute value are graphed. Specifically, features associated with a blue bar have reduced the classification score or the probability of being malignant. In contrast, the features associated with a red bar have increased the classification score or the probability that the lesion was malignant.

Figure 5 shows examples of correct classifications. The first example concerns a patient with a benign lesion, correctly classified by the system with a classification score of 0.16 (Figure 5A). The features that have contributed to lowering the classification score, pushing towards the ‘benign’ class, are the absence of the Color Score, CA125 equal to 14.1, the presence of shadows, the absence of ascites and age at diagnosis of 59 years, while the presence of personal history of breast cancer contributed to increasing the classification score, pushing towards the ‘malignant’ class. Substantially, the strength of the individual contributions generates the result of the classification score.

The second example instead concerns a patient with a metastatic lesion correctly classified as malignant by *model a*, according to the classification score of 0.71, then reevaluated by the second model (*model b*), and correctly classified as metastatic according to the classification score of 0.25 (Figure 5B). The features that contributed to increasing the classification score of the *model a*, pushing towards the ‘malignant’ class, were the very strong flow of the Color Score, the absence of both shadows and personal history of breast cancer. In contrast, the features contributing to the reduction of the classification score were CA125 equal to 33.7 and absence of ascites. Having been classified by the first model as malignant, the second model intervenes to establish whether or not the lesion is metastatic. The features that led to the ‘metastatic’ class are no family history of carcinoma, absence of free fluid, max diameter of the lesion equal to 43.0 mm plus personal history of breast cancer, and CA125.

Figure 6 instead shows examples of incorrect classifications. The first example shown in Figure 6A refers to a benign lesion erroneously classified as malignant with a classification score of 0.62 and then as nonmetastatic with a classification score of 0.11. Specifically, the features that have contributed to increasing the classification score of *model a*, pushing towards the ‘malignant’ class, the very strong flow of the Color Score, absence of Shadows, largest diameter of the lesion equal to 54 mm, and the menopausal status, while a CA125 equal to 9.9 and the absence of ascites push towards the benign class. These last two indicators, together with a not particularly high final classification score, could lead the operator to reevaluate the classification result of the automated system. In fact, the operator correctly classified this case. Furthermore, having been classified by the first model as malignant, the clinician also has information from the second model, which shows that in addition to the CA125, the absence of free fluid also lowers the probability that the lesion considered is metastatic.

A second example concerns a malignant patient incorrectly classified as benign with a classification score of 0.48 (Figure 6B). The features that contributed to assigning the ‘benign’ class are minimal flow of the Color Score, the presence of Shadows, and the absence of ascites. In contrast, the features that pushed towards the ‘malignant’ class were the age at diagnosis of 80 and CA125 equal to 3975.0, the latter parameter, which, together with the value of the classification score, borderline with respect to the cutoff used, could represent an alert for the clinician in evaluating the outcome suggested by the system. Indeed, the operator classified this case as malignant, although erroneously as metastatic.

Finally, a third example (Figure 6C) concerns a metastatic patient classified as nonmetastatic with a classification score of 0.66 but incorrectly classified as nonmetastatic with a score of 0.09. The features of *model b* that contributed to mis‐assigning the ‘metastatic’ class are age at diagnosis equal to 43, CA125 equal to 86.0, presence of a personal history of breast cancer, and a maximum diameter of 162 mm, parameters that could suggest a different diagnosis to the clinician. Indeed, this case was correctly classified by the operator.

## 4 DISCUSSION

Several studies demonstrated that the presence of solid tissue within an adnexal cyst on ultrasound evaluation is a suspicious finding for malignancy. Conversely, the absence of solid tissue in an adnexal mass is more likely to correlate with a benign lesion.^24^ Subsequent studies have further shown that a completely solid adnexal lesion carries a 65% risk of being a malignant lesion. However, there are solid ovarian lesions that correspond to benign histologies.^2^

In the largest case series of fibroma and fibrothecoma of the ovary published to date in the literature, the majority (75%) were solid tumors. They can exhibit round, oval, or lobulated borders with stripy shadows and generally show minimal or moderate vascularization.^25^, ^26^ Occasionally, they may be associated with fluid in the pouch of Douglas or ascites, leading to a misdiagnosis as malignancy.^26^

Frequently, invasive ovarian carcinoma is identified as a solid lesion on ultrasound imaging. Among the epithelial subtypes, the high‐grade serous carcinoma typically manifests as a solid mass (64%). Occasionally, multiple irregular cystic areas may be present within the solid components, likely related to necrosis, while calcifications are uncommon.^27^ Regarding ovarian clear cell carcinoma, the largest study on ultrasound examination of this histology reported that all masses contained solid components and 23.7% of them were completely solid masses. Typically, ovarian clear cell carcinoma presents as a large unilateral mass and is often diagnosed at an early stage.^28^ Large, unilateral masses are commonly observed in endometrioid ovarian cancers. When examined with ultrasound, this subtype of ovarian cancer is usually characterized by either multilocular‐solid cysts (48.1%) or solid masses (34.3%).^29^

Rare histologies of ovarian cancer may exhibit a distinct clinical presentation. Specifically, papillary borderline ovarian tumors (SSPBOTs), characterized as a rare morphologic variant of serous ovarian tumors, manifest solid tissue typically confined to the ovarian surface, surrounding normal ovarian parenchyma.^30^, ^31^

In the case of Sertoli and Sertoli‐Leydig and Leydig cell tumors, Demidov et al. reported that 96% of the 23 tumors in their case series contained solid components, with 70% being purely solid.^31^ Therefore, the identification of suspicious ultrasound features, even in masses of small dimensions, along with consideration of the patient's symptoms, could facilitate an accurate diagnosis and enable personalized treatment for the patient.^32^, ^33^

Metastatic ovarian tumors can appear as solid tumors on ultrasound evaluation.

Typically, these masses primarily comprise metastases of signet ring‐cell cancer originating from the stomach, appendix, or other sites. Additionally, they can arise from breast cancer, lymphoma, lung cancer, or melanoma. They present as bilateral solid tumors with a multi‐nodular appearance, moderate to intense vascularization, and may show findings of lead vessel or ring‐shaped vessels.^34^

The overlapping ultrasound features seen in different types of ovarian tumors make it challenging to evaluate a patient with a solid adnexal mass. This complexity has important implications for managing the patient effectively. Consequently, there is a growing demand for the development of an auxiliary tool that can assist clinicians in overcoming these challenges.^35^

The preliminary machine learning model proposed is based on a cascade‐model that allows the prediction, starting from the clinical characteristics and qualitative ultrasound indicators, first, if the lesion identified by the ultrasound examination is benign or malignant. Then if malignant, it also predicts whether it is metastatic or not.

The proposed model achieves encouraging performances that settle at 90.91% and 91.36%, respectively, for the benign versus malignant and nonmetastatic versus metastatic. Concerning the three‐class problem, the waterfall model achieves an accuracy of 86.36%. The latter performance is comparable with the operator's (86.36%) evaluated on the same real‐life sample, although the proposed model shows an accuracy of 77.78%. On the same sample, we also evaluated the accuracy of two tools used in clinical practice to discriminate benign versus malignant tumors, such as ADNEX and the Simple Rules, which reached 80.00% and 94.90%, respectively. However, it should be emphasized that the Simple Rules did not provide an evaluation for approximately 11% of the cases examined. Furthermore, as is known, the Simple Rules do not provide indications regarding the possibility of a metastatic lesion. However, the accuracy of ADNEX on the three‐class problem drops to 62.73%.

The tool proposed in this study provides additional information given by the Shapley values, which are expressed for each patient to identify the contribution of each feature to the classification outcome generated by the implemented machine learning model. Indeed, both on the first benign model versus malignant which on the second nonmetastatic malignant versus metastatic malignant, the system generates a graphical representation of how the values of the individual characteristics of a particular patient have contributed to the achievement of a specific classification score, that is, the assignment of a particular class. This aspect represents an added value for the clinical usability of a diagnostic system since it provides the clinician with a precise evaluation of how the automated system arrived at a given decision. The clinician has the possibility of evaluating which features contributed to achieving the classification result and the value of the variables themselves. In this way, he can evaluate the reliability of the suggestion provided according to his expertise and experience, and therefore accept or not the result provided by the machine.

Furthermore, a system capable of explaining in a more transparent way how it works and on which variables it bases its evaluations, allows the development of a sense of empathy and understanding in the logic of the machine, which is reflected in a relationship of natural trust towards the end user of the proposed technology.

Indeed, some work has recently been proposed to diagnose the malignancy of ovarian masses. The works in the literature aimed at establishing a diagnostic model of solid axial lesions use data of different nature.^36^ Even focusing on those developed from ultrasound images, the comparison is purely qualitative because the proposed models are radiomic‐based while our model is based on qualitative radiological indicators and clinical features.

Specifically, some of them use trained machine learning algorithms on radiomic features^37^, ^38^, ^39^ while others have used more sophisticated deep learning techniques.^40^, ^41^, ^42^ However, these models are trained to solve a binary problem (benign vs. malignant) therefore not comparable with our proposed model. Early and accurate prediction of metastatic status represents important information in planning the treatment pathway of the oncological patients. Furthermore, although the models proposed in the literature based on quantitative evaluation of ultrasound images achieve high performance above 90% accuracy, they do not provide the end user with an explicable tool.

To the best of our knowledge, there are no machine‐learning models in the literature based on clinical characteristics and qualitative radiological indicators. In addition, our study is the first work that attempts to design an explainable machine‐learning tool for the histological diagnosis of solid masses of the ovary. The proposed approach enables the clinician to make informed use of the prevision tool.

The work proposed here is preliminary work that needs to be validated and optimized on a wider case series. In fact, the limitations of this study are the limited case studies, especially with reference to the metastatic class, and the lack of an external validation cohort. Furthermore, some of the radiological indicators used are operator‐dependent assessments and this could represent a potential study bias. Future studies will be aimed at evaluating the robustness of the model with respect to variations in these indicators and at introducing any correction factors. However, it provides encouraging results and lays the foundations for future developments, which also see the integration of radiomic information with the clinical data and qualitative indicators used in this study.

## 5 CONCLUSION

The ultrasound characteristics of the different types of ovarian tumors often make the diagnosis of solid adnexal masses difficult. A correct diagnosis has important implications in defining the therapeutic treatment plan. Although there are diagnostic tools used in clinical practice, such as those proposed by IOTA models, the definition of a highly performant diagnostic tool that can support clinicians in this task is still an open challenge. In this study we proposed an explainable machine‐learning tool for the histological diagnosis of solid masses of the ovary starting on clinical characteristics and qualitative ultrasound indicators. The proposed model achieves high performance compared with the proposed state‐of‐the‐art tools and works. It also overcomes the black‐box concept associated with artificial intelligence tools by providing the end user, such as the clinician, with an explainable result.

We believe that the next approach lays the foundations for future validation and optimization studies for the definition of a tool that can be used in clinical practice.

## AUTHOR CONTRIBUTIONS

**Annarita Fanizzi:** Conceptualization (equal); formal analysis (equal); methodology (equal); software (equal); validation (equal); writing – original draft (equal); writing – review and editing (equal). **Francesca Arezzo:** Conceptualization (equal); resources (equal); writing – original draft (equal); writing – review and editing (equal). **Gennaro Cormio:** Project administration (equal); resources (equal); writing – original draft (equal); writing – review and editing (equal). **Maria Colomba Comes:** Methodology (equal); software (equal); writing – original draft (equal); writing – review and editing (equal). **Gerardo Cazzato:** Supervision (equal); writing – original draft (equal); writing – review and editing (equal). **Luca Boldrini:** Writing – review and editing (equal). **Samantha Bove:** Methodology (equal); writing – original draft (equal); writing – review and editing (equal). **Michele Bollino:** Data curation (equal); writing – review and editing (equal). **Anila Kardhashi:** Formal analysis (equal); writing – review and editing (equal). **Erica Silvestris:** Writing – original draft (equal); writing – review and editing (equal). **Pietro Quarto:** Data curation (equal); writing – review and editing (equal). **Michele Mongelli:** Data curation (equal); writing – review and editing (equal). **Emanuele Naglieri:** Writing – review and editing (equal). **Rahel Signorile:** Writing – review and editing (equal). **Vera Loizzi:** Writing – review and editing (equal). **Raffaella Massafra:** Conceptualization (equal); formal analysis (equal); methodology (equal); resources (equal); writing – original draft (equal); writing – review and editing (equal).

## FUNDING INFORMATION

This work was supported by funding from the Italian Ministry of Health “5 per 1000” project (Deliberation n. 655/2022).

## CONFLICT OF INTEREST STATEMENT

The authors declare no conflict of interest.

## CONSENT

Written informed consent for participation was not required for this retrospective observational study in accordance with the institutional requirements.

## Data Availability

The data presented in this study are available on request from the corresponding author. The data are not publicly available because are proper of the Azienda Ospedaliera Policlinico Consorziale‐University of Bari.
